# Cytomegalovirus Infection May Contribute to the Reduced Immune Function, Growth, Development, and Health of HIV-Exposed, Uninfected African Children

**DOI:** 10.3389/fimmu.2016.00257

**Published:** 2016-06-30

**Authors:** Suzanne Filteau, Sarah Rowland-Jones

**Affiliations:** ^1^Department of Population Health, London School of Hygiene & Tropical Medicine, London, UK; ^2^Nuffield Department of Medicine, Oxford University, Oxford, UK

**Keywords:** HIV-exposed uninfected, cytomegalovirus, children, Africa, immune function

## Abstract

With increasing access to antiretroviral therapy (ART) in Africa, most children born to HIV-infected mothers are not themselves HIV-infected. These HIV-exposed, uninfected (HEU) children are at increased risk of mortality and have immune, growth, development, and health deficits compared to HIV-unexposed children. HEU children are known to be at higher risk than HIV-unexposed children of acquiring cytomegalovirus (CMV) infection in early life. This risk is largely unaffected by ART and is increased by breastfeeding, which itself is critically important for child health and survival. Early CMV infection, namely *in utero* or during early infancy, may contribute to reduced growth, altered or impaired immune functions, and sensory and cognitive deficits. We review the evidence that CMV may be responsible for the health impairments of HEU children. There are currently no ideal safe and effective interventions to reduce postnatal CMV infection. If a clinical trial showed proof of the principle that decreasing early CMV infection improved health and development of HEU children, this could provide the impetus needed for the development of better interventions to improve the health of this vulnerable population.

## Health Problems of HIV-Exposed, Uninfected African Children

HIV-exposed, uninfected (HEU) children have always constituted the majority of children born to HIV-infected mothers, but until recently the focus has been on preventing mother-to-child HIV transmission and treating HIV-infected children, so HEU children have received little attention. Preventing mother-to-child HIV transmission remains crucial and the situation is improving with increasing provision of antiretroviral therapy (ART). As a result of decreasing infant HIV infection, the numbers of HEU children are rising and in high HIV prevalence countries of Southern Africa may account for up to 30% of births (1, 2). Thus, any health problems HEU children experience are of immense public health importance.

Even when HIV-infected mothers are provided with ART, their HEU infants experience numerous health and development problems (3). Mortality of HEU children is increased over that of their HIV-unexposed counterparts (4). Compared with HIV-unexposed children, HEU children have lower birth weight (5), impaired early growth (6), impaired psychomotor and cognitive development (7), and immune abnormalities (8), presumably resulting from stressors during *in utero* or early postnatal life. The underlying causes of these impairments in HEU infants are probably multifactorial and include exposure to HIV itself (9, 10), exposure to ART (5), increased exposure to infections within the household (11), and the consequences of being born into an HIV-afflicted household, with impact on socioeconomic status and child care quality (4). The relative contribution that these different factors make to the health deficits of HEU children are likely to differ in different populations and all should be addressed.

Here, we review the evidence in support of the hypothesis that one potential cause, early cytomegalovirus (CMV) infection, which appears widespread across sub-Saharan Africa, is an important contributor to impaired health and development of both HIV-infected and HEU children in many African populations, and increases the risk of postnatal HIV infection. The synergism between HIV and CMV coinfection in African adults and children has been recently reviewed (12), so this article will focus on HEU children, in particular those in Africa where the burden of both CMV and HIV infection is highest.

## Early CMV Infection among HIV-Exposed Infants

Most African infants acquire CMV infection during infancy (13, 14). The risk of congenital CMV infection was higher among infants of HIV-infected than uninfected Zambian mothers (15). Increased infections with maternal HIV exposure could result from maternal CMV reactivation, which is common in HIV-infected women, particularly with low CD4 counts; CMV was detected in the serum and cervix of, respectively, 4.8 and 66% ART-treated HIV-positive pregnant Kenyan women (16). Reduced placental transfer of maternal antibodies in HIV-infected women (8) may also contribute to the increased risk of early CMV infection of their infants (17). CMV infection mainly occurs in African infants during the early postnatal period and a large proportion of CMV transmission is through breastfeeding. A secondary analysis of a randomized controlled trial of breastfeeding versus formula-feeding by HIV-infected Kenyan women in the pre-ART era found that breastfeeding was associated with a significantly increased proportion of infants CMV-infected by age 1 year (89 versus 69% in the formula group), as well as earlier median acquisition of CMV infection (4.26 versus 9.87 months) (18). Nevertheless, as previously shown for HIV transmission in the Kenyan trial (19) as well as in other studies, breastfeeding is important for child survival and health, so switching from breastfeeding is not a feasible way to reduce transmission of CMV.

Currently, the World Health Organization recommends provision of ART during pregnancy and lactation as the best way of promoting HIV-free survival of infants (20). Although this protocol is becoming standard across Africa and is effective at decreasing HIV transmission, it does not appreciably alter the rate of African infant CMV acquisition. The rate of congenital CMV infection in South African infants did not differ according to whether their mothers had taken during pregnancy zidovudine only or triple-drug ART (21). Breast milk CMV load was similar among Malawian women who had started ART for their own health and women not on ART, even though the ART-treated women had lower plasma and milk HIV levels and both groups had similar CD4 counts (22). A recent study of American women whose ART did or did not include the protease inhibitor, nelfinavir, which has some ability to inhibit CMV replication, found no protection of nelfinavir against congenital CMV infection (23).

## Effects of Early CMV Infection on Health of HEU Children

Most concerns about CMV among non-HIV-exposed infants, particularly in the developed world, relate to congenital CMV. Congenital CMV arising from maternal primary or reactivated CMV infection during pregnancy is associated with intrauterine growth retardation and neurological, hearing, and vision impairments in the infected infant (24, 25). In particular, 25–50% of infants diagnosed at birth with symptomatic congenital CMV (and 10% of asymptomatic congenital infections) suffer from hearing loss (26), which is often not detected until later in infancy (27). A study of CMV screening at birth for over 300 infants born to HIV-infected mothers found that, although the rates of congenital CMV infection were low (10 infants, 3%), they were higher than usually observed in HIV-unexposed infants; moreover, HEU infants with asymptomatic congenital CMV had significantly lower birth weight and lower gestational age compared with infants who were CMV-uninfected at birth (28).

Whether the early postnatal CMV infection, which is common among HEU African infants, also has adverse effects on health needs to be considered. In the pre-ART era, serum CMV in HIV-infected women at delivery was associated with almost 10-fold increase in maternal death and a fourfold increase in mortality of their HIV-infected infants by age 2 years; this remained statistically significant even after adjustment for maternal CD4 count, HIV viral load, and death (29). Early CMV infection also results in a 2.5-fold to 4-fold increased risk of postnatal HIV infection (29–31), and CMV coinfection leads to more rapid infant HIV disease progression (29, 32). It is worth noting that several reports have indicated that children of HIV-positive mothers, both infected (33) and HEU (34), are more likely than their unexposed counterparts to have impaired hearing; although other mechanisms, including exposure to HIV itself, may have contributed to deafness in these children, it could well be an indication of congenital (or even early) CMV infection that was not diagnosed at birth. Because the hearing loss associated with congenital CMV infection is often not diagnosed until later childhood (particularly at school entry), it would potentially be worth instituting formal monitoring of the hearing of HEU infants to avoid the development and educational problems often faced by children with undiagnosed deafness.

Surviving HEU children exhibit impairments including poor infant growth; (6, 22) reduced psychomotor development; (7) and immunological abnormalities (see below). In observational studies, CMV infection was associated with poorer infant growth (13, 22) and delayed psychomotor development (13). In a study in Malawi, breast milk CMV DNA viral load had a stronger negative association with infant growth than did milk HIV RNA load; the largest effects occurred in the first 4–6 weeks of the infant’s life, suggesting that prenatal or early postnatal exposure to CMV is key (22).

## Immunological Consequences of Early CMV Infection in HEU Infants

In older Europeans, CMV infection is associated with frailty and increased mortality, predominantly through cardiovascular disease, with an estimated 47% increased risk of death compared to uninfected people in a large UK population of people aged 65 years and over (35). CMV seropositivity in the elderly is linked with a number of immunological features that include large expansions of CMV-specific T-cells with reduced naive T-cell numbers (36), raised C-reactive protein (37), and impaired immune responses to influenza vaccination (38). In contrast, CMV is acquired very early in life in Africa, with the majority of infections occurring during infancy [85% infection at one year in a Gambian birth cohort (14)]: the long-term consequences of early CMV acquisition in developing countries are not known.

The large CMV genome contains multiple genes linked with immune evasion [including the down-regulation of class I Histocompatibility Leukocyte Antigen (HLA) molecules, interference with antigen processing, suppression of T-cell proliferation, and production of a viral version of the immunosuppressive chemokine interleukin-10], which could potentially affect immune responses to other pathogens, although this has yet to be unequivocally demonstrated. Infant CMV infection is associated with profound changes in the phenotype of CD8^+^ and CD4^+^ T-cells, which show increased levels of activation and differentiation (39–41) similar to those described in older Europeans; however, there is no evidence of CMV-associated immune impairment in healthy infants, and vaccine responses in HIV-unexposed infants appear to be unaffected by CMV status, at least in the short term (42, 43). The overall impact of CMV infection on vaccine responses is not well understood: in young adults, CMV infection is associated with enhanced antibody and T-cell responses to influenza vaccination (44), but in a different adult cohort CMV seropositivity was linked with reduced Natural Killer (NK) cell responses to pertussis and H1N1 influenza A antigens (45). In older adults, CMV seropositivity is associated with significantly decreased CD4 T-cell responses to influenza A core antigens (38).

Although HEU infants generally show robust vaccine responses, at least in the short term (46), CMV infection in HEU infants born to Zambian HIV-infected mothers was associated with impaired polio vaccine responses (47). The majority of the immunological perturbations described in HEU infants, including the expansion of memory T-cell subsets, reduction in naive T-cells, and heightened immune activation [reviewed in Ref. (8)], are observations consistent with and potentially explained by CMV infection occurring earlier in HEU children than in the control populations studied. Few of the published studies of the immunology of HEU infants have accounted for infection with CMV [or other herpes viruses with known impact on the immune system, such as Epstein–Barr virus (EBV)].

## Impact of Maternal CMV Infection on the Placenta

As discussed earlier, it is not yet clear whether early CMV infection has an adverse effect on infant health in the absence of HIV exposure, immunosuppression, or congenital infection. Before the advent of CMV screening for infant blood transfusion, postnatal transmission of CMV to premature and low birth weight infants was associated with a range of symptoms, including fever, pneumonia, hepatosplenomegaly and hepatitis, and a high risk of death, but these children could be regarded as immunosuppressed (48). However, it may also be worth considering that some of the abnormalities described in HEU children imply that the fetus faced challenges in the prenatal environment that affected the placenta, notably the observation of marked stunting that cannot be corrected by supplementary feeding in the first year of life (6) and the reduced transfer of maternal antibodies to the infant (49, 50), which requires an active process by the placenta in the last trimester. CMV can infect the placenta without infecting the infant, particularly in women with reactivated rather than primary CMV infection. For example, in a detailed study of the role of CMV in seven cases of intrauterine growth restriction, five were associated with CMV infection, of which three had evidence of reactivated infection and did not lead to *in utero* infection of the infant (24). Nevertheless, there was evidence of CMV proteins in placental tissue and associated pathology, such as edema and leukocyte infiltration, which suggests that CMV infection of the placenta can lead to impaired fetal growth without necessarily resulting in congenital infection. It was recently reported that CMV infection of trophoblast progenitor cells *in vitro* interferes with the early steps of the growth of placental villi and could therefore affect placental maturation and transport functions (51).

## Approaches to Decreasing Early CMV Infection of HEU Infants

Although the observational data linking early CMV infection with the poorer health and development of HEU African infants are strong, a controlled clinical trial is needed before we can assume that CMV infection causes these impairments. There is currently no ideal intervention, effective against CMV infection, safe for pregnant or lactating women and their infants, and feasible for use in Africa. A recent trial with a low dose (500 mg twice daily) of valacyclovir (GlaxoSmithKline) had no effect on the timing of infant CMV infection or breast milk CMV viral load; higher doses of the drug, as used to prevent CMV infection among transplant patients, might have had more effect but were considered unrealistic for pregnant and lactating women (17). Valganciclovir (Hoffman-La Roche) has greater anti-CMV effect than valacyclovir, has been used postnatally for congenital CMV infection (25), and led to reduced T cell activation among HIV-infected adults (52). However, a recent trial comparing 6 months versus the standard 6 weeks of valganciclovir for congenital CMV infection found side effects in a proportion of infants: 19% of infants had grade 3–4 neutropenia during the first 6 weeks of life that in three of 109 infants necessitated temporary suspension of treatment (25). A trial of hyperimmune globulin to prevent congenital CMV infection in infants of women with primary CMV infection found no significant decrease in congenital CMV infection, similar clinical outcomes among congenitally infected infants, and a higher rate of obstetric adverse events among the women treated with globulin compared to those given placebo (53).

There are a number of CMV vaccines under development [recently reviewed in Ref. (54)]. These vaccines have largely been developed with the aim of preventing primary CMV infection in women of child-bearing age in developed countries. The risk of congenital CMV infection with primary infection during pregnancy is around 40%, whereas only 1–2% of already-infected women transmit CMV to their infants. Current candidate CMV vaccines predominantly aim to elicit neutralizing antibodies, using either the CMV surface glycoprotein (gB) (55), or the recently described pentameric complex (gH/gL/UL128-UL130-UL131) (56), which is required for CMV entry into epithelial cells (57). The induction of a strong, high avidity, neutralizing antibody response may also reduce congenital infection in infants of women with preexisting infection, but in HIV-infected women this could be impaired by the reduction in placental transfer of maternal antibodies that occurs during pregnancy. Other CMV candidate vaccines focus on the induction of T-cell immunity, which may have more value in controlling CMV reactivation in pregnancy and HIV infection. A phase III trial is underway in seropositive transplant recipients of a bivalent CMV DNA vaccine, expressing both gB and pp65, the main target of the T-cell response (58). In the long term, a vaccine that effectively prevents both congenital and early CMV transmission may have a major impact on the health of infants born to HIV-infected mothers in sub-Saharan Africa.

## Conclusion and Recommendations

The health and development problems of HEU infants are almost certainly multifactorial, and this review focuses on one of these factors, early CMV infection. Evidence for the potential importance of early CMV infection as a contributor to poorer outcomes in HEU infants is increasing but is not yet definitive. The field now requires a clinical trial of a safe and effective anti-CMV intervention in order to move forward. At this juncture, a trial of a currently available intervention, such as valganciclovir given to lactating women or their infants to prevent early infant infection, would be of considerable benefit in resolving these issues. If such an intervention showed proof of principle that delaying CMV infection in HEU infants benefited their health without unacceptable adverse effects, this would provide impetus for the design of safer and more efficacious CMV interventions for this large and vulnerable population. Even a safe drug would increase the already large pill burden in HIV-infected lactating women or their infants so qualitative and programmatic research would be needed to ensure that an anti-CMV treatment was acceptable as well as efficacious in relevant populations. However, there is a precedent for adding additional drugs to ART care, specifically the widespread use of prophylactic Cotrimoxazole, suggesting that an effective anti-CMV treatment could well be similarly acceptable.

## Author Contributions

SF and SRJ contributed equally to the conception and writing of this work.

## Conflict of Interest Statement

The authors declare that the research was conducted in the absence of any commercial or financial relationships that could be construed as a potential conflict of interest.
